# Three new RelE-homologous mRNA interferases of *Escherichia coli* differentially induced by environmental stresses

**DOI:** 10.1111/j.1365-2958.2009.06969.x

**Published:** 2009-12-03

**Authors:** Mikkel Christensen-Dalsgaard, Mikkel Girke Jørgensen, Kenn Gerdes

**Affiliations:** Centre for Bacterial Cell Biology, Institute for Cell and Molecular Biosciences, Medical School, Newcastle UniversityNewcastle NE2 4HH, UK.

## Abstract

Prokaryotic toxin – antitoxin (TA) loci encode mRNA interferases that inhibit translation, either by cleaving mRNA codons at the ribosomal A site or by cleaving any RNA site-specifically. So far, seven mRNA interferases of *Escherichia coli* have been identified, four of which cleave mRNA by a translation-dependent mechanism. Here, we experimentally confirmed the presence of three novel TA loci in *E. coli*. We found that the *yafNO, higBA (ygjNM)* and *ygiUT* loci encode mRNA interferases related to RelE. YafO and HigB cleaved translated mRNA only, while YgiU cleaved RNA site-specifically at GC[A/U], independently of translation. Thus, YgiU is the first RelE-related mRNA interferase that cleaves mRNA independently of translation, *in vivo*. All three loci were induced by amino acid starvation, and inhibition of translation although to different degrees. Carbon starvation induced only two of the loci. The *yafNO* locus was induced by DNA damage, but the transcription originated from the *dinB* promoter. Thus, our results showed that the different TA loci responded differentially to environmental stresses. Induction of the three loci depended on Lon protease that may sense the environmental stresses and activate TA loci by cleavage of the antitoxins. Transcription of the three TA operons was autoregulated by the antitoxins.

## Introduction

Prokaryotic toxin – antitoxin (TA) loci encode toxins whose ectopic production induces cell killing or inhibits cell growth and antitoxins that counteract the toxins (Gerdes *et al.*, 2005). TA loci are present in almost all free-living prokaryotes, often in surprisingly high numbers (Pandey and Gerdes, 2005). For example, *Mycobacterium tuberculosis* has more than 60 TA loci while another pathogen, *Photorhabdus luminisens* has at least 59 and the obligate chemolithotroph *Nitrosomonas europaea* has at least 51 (Pandey and Gerdes, 2005; Jørgensen *et al.*, 2009). By contrast, obligatory intracellular organisms have few or none. The model organism *Escherichia coli* also encodes a large number of TA loci that were divided into two types based on the molecular nature of the antitoxin (Hayes, 2003). Type I TA loci, such as *hok*/*sok*, *ldr*, *symE* and *tisAB*, encode antisense RNAs that inhibit translation of the toxin-encoding mRNAs (Gerdes and Wagner, 2007; Waters and Storz, 2009) while type II loci encode protein antitoxins that neutralize the toxins by direct protein–protein contact. So far, seven type II TA loci type have been identified in *E. coli* (*relBE*, *dinJ yafQ*, *yefM yoeB*, *prlF yhaV*, *mazEF*, *chpSB* and *hicAB*) (Masuda *et al.*, 1993; Gotfredsen and Gerdes, 1998; Schmidt *et al.*, 2007; Jørgensen *et al.*, 2009; Prysak *et al.*, 2009). Interestingly, all the type II loci of *E. coli* encode mRNA interferases whose ectopic overproduction inhibits translation by mRNA cleavage. Recently, a third type of TA loci was identified. In this case, the antitoxin is a small *cis*-encoded RNA that inhibits toxin activity by direct molecular interaction (Blower *et al.*, 2009).

The biological function of mRNA interferase-encoding TA loci has been debated (recently reviewed in Magnuson, 2007; Hayes and Low, 2009; Van Melderen and Saavedra De Bast, 2009). Some scientists observed that mRNA interferases, such as MazF of *E. coli*, induced cell killing and therefore suggested that mRNA interferases function to mediate programmed cell death (Engelberg-Kulka *et al.*, 2006). However, a more common view is that mRNA interferases function in growth rate control or persister cell formation (see the excellent recent review by Hayes and Low, 2009 for an in-depth discussion). These two models, which are not mutually exclusive, are consistent with observations made by many laboratories, namely that ectopic production of mRNA interferases is bacteriostatic rather than bacteriocidal (Pedersen *et al.*, 2002; Christensen *et al.*, 2003; Christensen-Dalsgaard and Gerdes, 2006; Budde *et al.*, 2007; Schmidt *et al.*, 2007; Fu *et al.*, 2009; Jørgensen *et al.*, 2009). Transcription of the *relBE*, *mazEF* and *hicAB* loci is strongly induced by conditions of amino acid (aa) starvation (Christensen *et al.*, 2001; 2003; Jørgensen *et al.*, 2009). It is well established that aa starvation does not induce cell death in *E. coli* standard strains (Cashel *et al.*, 1996; Christensen *et al.*, 2001; Traxler *et al.*, 2008). In this connection we find it important to distinguish between *E. coli* and other organisms. This is because MazF of *Myxococcus xanthus* was produced during spore development and induced massive lysis of the cells that did not develop into spores (Nariya and Inouye, 2008). MazF_Mx_ homologue is not located adjacent to a *mazE*-like gene that encodes an antitoxin. Rather, MazF_Mx_ is regulated by a key developmental factor encoded elsewhere in the *M. xanthus* chromosome. Thus in this special case, a MazF homologue has been recruited to function in a developmental programme.

In case of *relBE*, the induction mechanism has been investigated in detail. The RelB antitoxin autoregulates transcription of the *relBE* operon and RelE acts as a corepressor such that transcription during steady state cell growth is highly repressed (Gotfredsen and Gerdes, 1998; Overgaard *et al.*, 2009). Moreover, RelB is degraded by Lon (Christensen *et al.*, 2001). Thus, when aa starvation ensues and the global translation rate is reduced by ≈10-fold, RelB is degraded and transcription of *relBE* increases dramatically (20–30-fold). This increase is due not only to degradation of RelB but also to the fact that RelE in excess of RelB disrupts the RelBE–operator complex and thereby stimulate transcription (Overgaard *et al.*, 2008). Thus, nutritional deprivation and other stressful conditions result in activation of RelE and hence reduce the global rate of translation by mRNA cleavage (Christensen *et al.*, 2001; Christensen and Gerdes, 2003). It should be emphasized that *relBE* reduced translation significantly during aa starvation but did not induce cell stasis, as is often surmised in the literature. Induction of *relBE* locus transcription in its native context reduces translation to a basal and balanced level that is reached rapidly after onset of starvation (10–15 min). During strong aa starvation (induced by serine hydroxamate, SHX) the global level of translation is *c*. 100% higher in a *relBE* deletion strain than in a wild-type strain (Christensen *et al.*, 2001). However, some RelB variants that are metabolically destabilized due to single aa changes confer hyper-induction of RelE and a complete shut-down of translation during aa starvation (Christensen and Gerdes, 2004), consistent with the induction model described above. However, even in this case the effect of RelE activation is bacteriostatic, not bacteriocidal.

Based on their mechanism of action, the mRNA interferases of *E. coli* can be divided into two types, those that cleave mRNA at the ribosomal A site (RelE, YafQ and YoeB) and those that cleave mRNA independently of the ribosome (MazF, ChpBK, HicA) (Pedersen *et al.*, 2003; Zhang *et al.*, 2003; 2005a; Christensen-Dalsgaard and Gerdes, 2008; Jørgensen *et al.*, 2009; Prysak *et al.*, 2009; Zhang and Inouye, 2009). Of the ribosome-dependent mRNA interferases, RelE has been particularly well described. RelE associates with the ribosomes (Galvani *et al.*, 2001) and cleaves mRNA positioned at the A site between the 2nd and 3rd nucleotides of the codon, both *in vitro* and *in vivo* (Christensen and Gerdes, 2003; Pedersen *et al.*, 2003). RelE can cleave within all codons, including stop codons, but have sequence preferences. For example, codons with a G at the 3rd position are cleaved much more efficiently than codons with a G at the 2nd position. RelE exhibits an incomplete RNase Sa fold – that is, it lacks the catalytic aa triad present in homologous RNases (Li *et al.*, 2009). This fact is consistent with the observation that RelE does not cleave naked RNA *in vitro*. YoeB of *E. coli* exhibits a similar fold, but contains the catalytic triad and consistently, YoeB cleaves naked RNA *in vitro* although with low efficiency (Kamada and Hanaoka, 2005). However, recent evidence suggests that YoeB, like RelE, cleaves mRNA by a ribosome-dependent mechanism *in vivo* (Christensen-Dalsgaard and Gerdes, 2008; Li *et al.*, 2009; Zhang *et al.*, 2009). YafQ, another RelE homologue of *E. coli*, has similarly been shown to cleave naked RNA *in vitro* whereas cleavage *in vivo* was dependent on translation of the target RNA (Prysak *et al.*, 2009).

The first ribosome-independent mRNA interferase to be described was MazF of *E. coli* (Zhang *et al.*, 2003). MazF cleaved translated and non-translated RNAs at ACA sites. Early investigations from our laboratory suggested that MazF activity depended on translation of the substrate RNA (Christensen and Gerdes, 2003) whereas other groups clearly did not observe such dependency (Zhang *et al.*, 2005b). This puzzle was recently resolved by the observation that translation stimulates MazF cleavage seemingly by unfolding the secondary structure of the target RNA (Christensen-Dalsgaard and Gerdes, 2008).

One important objective in the TA field is to determine the biological function of TA loci. To firmly address this question it is crucial to know the exact number of redundant systems in the organisms of interest. To that end we wish to identify all TA loci present in the model organism *E. coli*. A recently developed Web-based algorithm, RASTA-Bacteria was previously used to suggest additional potential TA loci in *E. coli* (Sevin and Barloy-Hubler, 2007). Here we validate and characterize three of these new TA loci in *E. coli, yafNO*, *ygjNM* and *ygiUT*, and demonstrate that they all encode mRNA interferases that are induced by aa starvation and other stressful conditions. Two loci encode ribosome-dependent mRNA interferases while the third cleaves mRNA as an RNA restriction enzyme, independently of translation. Interestingly, the three TA loci responded differentially to various environmental stresses, indicating that TA loci may serve to restrict translation during different adverse growth conditions.

**Fig. 1:**
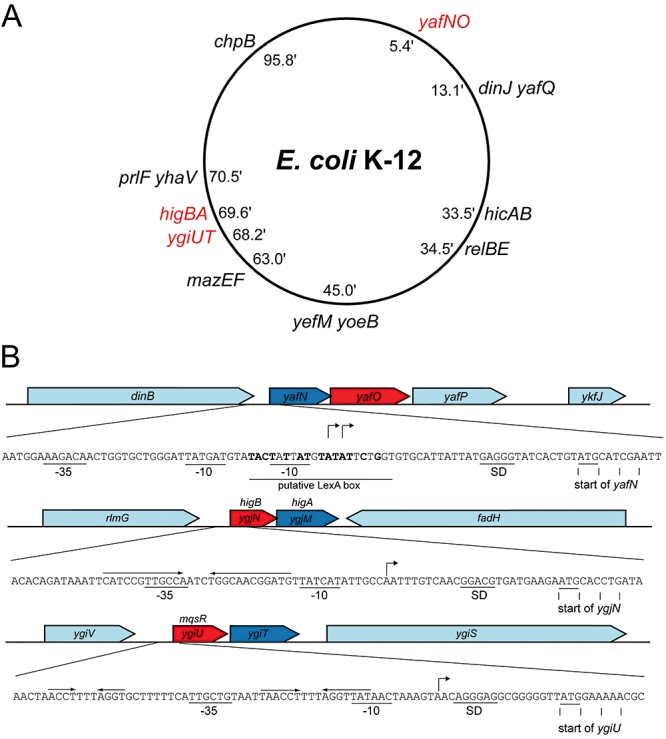
Three new toxin-antitoxin loci of *E. coli*. A. Chromosomal positions of the seven well-characterized toxin-antitoxin loci of *E. coli* K1: *relBE*, *yefM yoeB*, *mazEF*, *prlF yhaV*, *chpSB* and *dinJ yafQ* (black text) and the three new TA loci *yafNO*, *higBA* (*ygjNM*) and *ygiUT* (red text). All 10 loci encode mRNA interferases. B. Genetic overview of the three new TA loci, *yafNO*, *higBA* (*ygjNM*) and *ygiUT* and the flanking DNA regions. Toxin genes are coloured read, antitoxin genes are blue. The promoter regions of the TA operons are shown as a blow up below each gene pair. Transcriptional start sites were mapped (Fig. 5) and shown as broken arrows pointing rightward. The start sites of the first gene in the TA operons are also shown. Putative −10, −35 and Shine and Dalgarno (SD) sequences are indicated. Inverted repeats that are putative protein binding sites are shown as opposing arrows. The *yafNO* promoter has a putative LexA box. Bases that conform to the canonical LexA box are shown in bold.

Secondary structure predictions according to PSIPRED (McGuffin *et al.*, 2000) suggested that toxins HigB/YgjN, YgiU/MqsR and YafO all belong to the RelE super-family of RNases (Fig. S1). *higBA* loci encode RelE-homologous mRNA interferases (Pandey and Gerdes, 2005; Christensen-Dalsgaard and Gerdes, 2006). The sequence similarity with RelE is usually modest and we defined *higBA* loci as *relBE* loci with an inverted gene order – that is, the toxin is encoded by the first gene of the TA operon. According to the EcoCyc database (Keseler *et al.*, 2009), the *ygjNM* genes have not been investigated before. Based on the experimental evidence presented here showing that YgjN is functionally related to RelE, the fact that the *ygjNM* locus has an ‘inverted’ gene order (Fig. 1), and the strong resemblance of the predicted secondary structures between RelE and YgjN, we suggest that *ygjNM* be renamed *higBA*. We also believe that the *ygiUT (mqsR/ygiT*) locus fits the criteria for being named a *higBA* locus. However, as its original name has been used several times in the literature we chose to keep the original nomenclature.

**Fig. 5:**
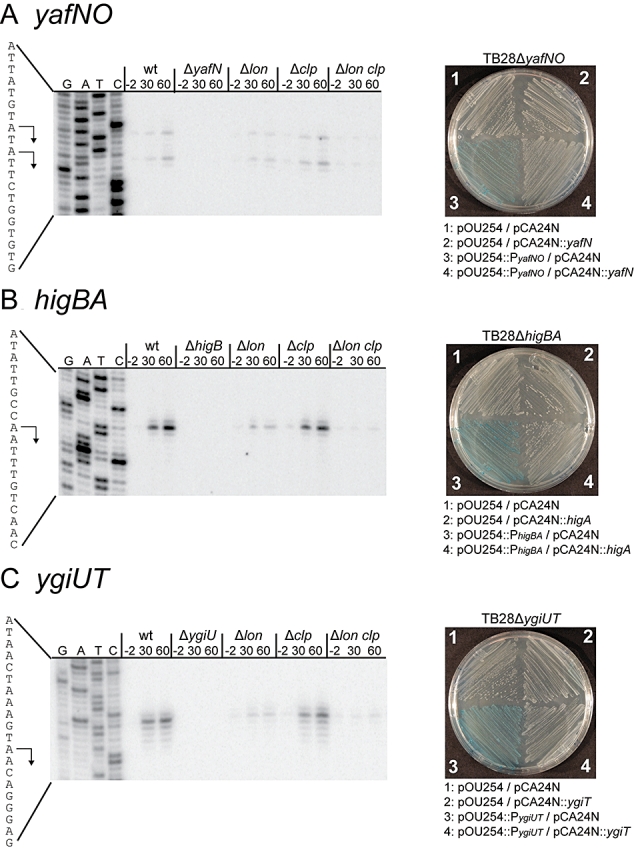
Mapping of the *yafNO, higBA* and *ygiUT* promoters. Left panels: Primer extension analysis on total RNA purified from cells of either wild-type MG1655 (wt) or Δ*yafN*, Δ*higB*, Δ*ygiU*, Δ*lon*, Δ*clp* or Δ*lon clp*. Cells were grown exponentially in LB medium and at time zero protein synthesis was inhibited with chlorampenicol (50 μg ml^−1^). The ^32^P-labelled primers, yafN PE1, ygjN PE1 and ygiU PE1 were used to map the 5′ ends of *yafNO* (A), *higBA* (B) and *ygiUT* (C) mRNAs respectively. The putative transcriptional start sites are marked with arrows is the text panel next to each gel. Right panels: DNA fragments carrying the expected promoter sequences were inserted upstream of *lacZ* in transcriptional fusions. Cells of TB28Δ*yafNO* (A), TB28Δ*higBA* (B) or TB28Δ*ygiUT* (C) were transformed with empty pOU254 or derivatives containing promoter fusions to *lacZ* and empty pCA24N or derivatives carrying the relevant antitoxins under the control of the IPTG-inducible T5-lac promoter. Cells were plated on LA containing Amp (30 μg ml^−1^), Cml (50 μg ml^−1^), Xgal (40 μg ml^−1^) and IPTG (0.5 mM).

## Results

### Three novel toxin-antitoxin gene pairs of *E. coli*

**Fig. 2:**
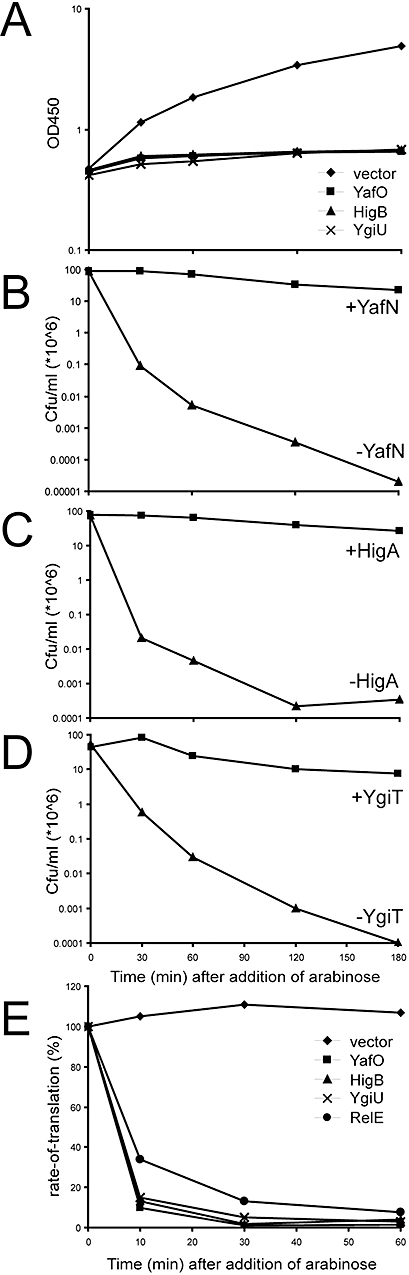
*yafNO, higBA* and *ygiUT* are bona fide toxin-antitoxin loci. A. Growth of *E. coli* MG1655 (wt) carrying one of the plasmids, pBAD33 (vector), pMCD3306 (pBAD::SD::*yafO*), pBAD3310 (pBAD::SD::*higB*) or pMCD3312 (pBAD::SD::*ygiU*). Cells were grown exponentially in LB medium at 37°C. At time zero (OD_450_ ≈ 0.5), arabinose (0.2%) was added to induce transcription of the toxin genes. B–D. Cells of MG1655/pMCD3306 (pBAD::SD::*yafO*)/pMCD2202 (P_A1/O3/O4_::SD::*yafN*) (B), MG1655/pMCD3310 (pBAD::SD::*higB*)/pMCD2205 (P_A1/O3/O4_::SD::*higA*) (C) and MG1655/pMCD3312 (pBAD::SD::*ygiU*)/pMCD2207 (P_A1/O3/O4_::SD::*ygiT*) (D) were grown exponentially in LB medium at 37°C. Transcription of the toxin genes was induced by addition of arabinose (0.2%) at time zero and cells plated on LA plates with IPTG (2 mM) (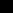) or without IPTG (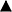) to induce transcription of the antitoxin genes. The number of colony-forming units ml^−1^ was calculated for each of the indicated time points. E. Cells of *E. coli* MG1655 carrying pBAD33 (vector), pMG3323 (pBAD::*relE*), pMCD3306 (pBAD::SD::*yafO*), pBAD3310 (pBAD::SD::*higB*) or pMCD3312 (pBAD::SD::*ygiU*) were grown exponentially in M9 minimal medium and the transcription of the toxins induced at time zero with arabinose (0.2%). Samples were taken at the indicated time points, and protein synthesis was measured by pulse-labelling cells for 1 min with ^35^S-methionine and chased for 10 min with cold methionine. The pre-incubation rates were set to 100%.

To investigate whether the loci *yafNO*, *higBA (ygjNM)* and y*giUT* of *E. coli* K12 encode functional TA systems, the putative toxin genes (*yafO*, *higB* and *ygiU*) and antitoxin genes (*yafN*, *higA* and *ygiU*) were cloned on separate plasmids. The toxin genes were inserted downstream of the arabinose-inducible promoter of pBAD33 and the putative antitoxin genes were inserted into pNDM220, which contains a strong, synthetic LacI-regulated promoter (pA1/O3/O4). A series of growth experiments were carried out in strains carrying cognate plasmid pairs (Fig. 2A–D). To prevent activation of endogenous TA loci as observed previously (Garcia-Pino *et al.*, 2008; Winther and Gerdes, 2009), all experiments were carried out in SC301467, a strain that lacks five TA loci (*relBE*, *yefm yoeB*, *dinJ yafQ*, *mazEF* and *chpSB*) (Christensen *et al.*, 2004). As seen, transcriptional induction of the three putative toxin genes led to an almost immediate inhibition of cell growth in all three cases (Fig. 2A). Furthermore, a significant decrease in the number of colony-forming units was observed on plates without isopropyl β-D-1-thiogalactopyranoside (IPTG) (Fig. 2B–D; −IPTG). However, cells of induced cultures could be almost completely resuscitated when plated on solid medium containing IPTG that induced the putative antitoxin genes (Fig. 2B–D; +IPTG).

Including these new genes, *E. coli* K-12 has 10 TA loci all encoding mRNA interferases. The genomic locations of the 10 loci are shown in Fig. 1A. The *yfeCD* and *ydaST* loci were also predicted to be TA loci by the RASTA-Bacteria algorithm (Sevin and Barloy-Hubler, 2007), but we did not find evidence that these two loci encode functional toxins and antitoxins (data not shown).

### The toxins inhibit translation by targeting mRNA

**Fig. 3:**
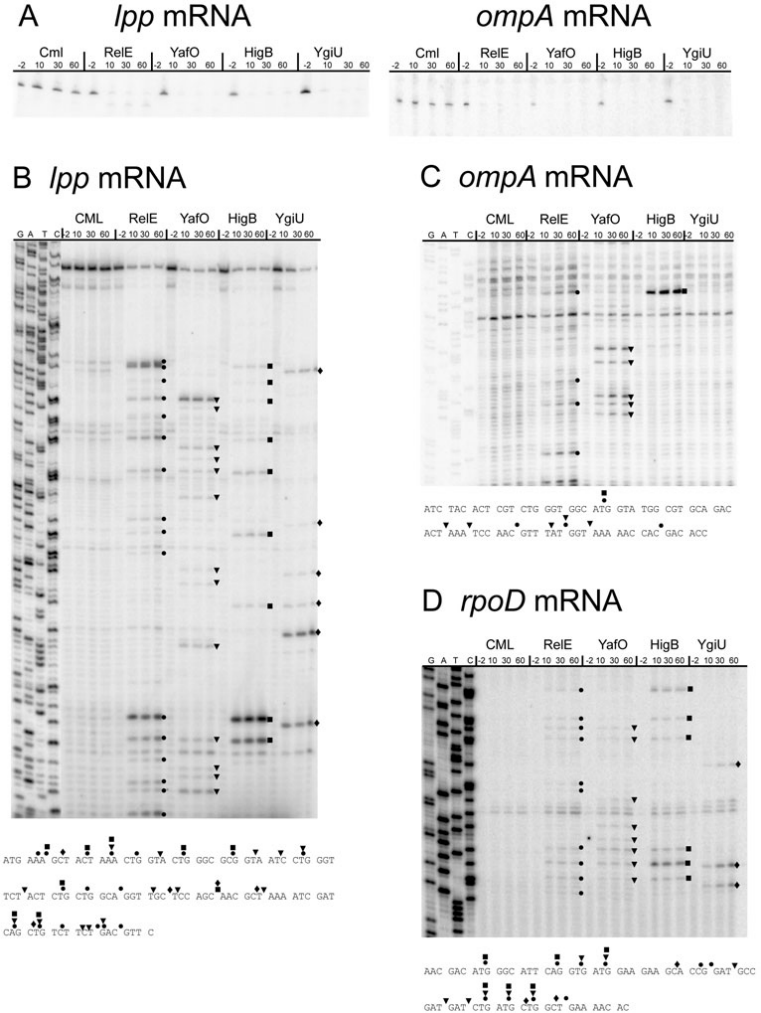
Ectopic induction of *yafO*, *higB* and *ygiU* confers mRNA cleavage. A. Northern analysis of the *lpp* and *ompA* mRNAs using specific ^32^P-labelled ribonucleoacid probes. Primer extensions on *lpp* (B), *ompA* (C) and *rpoD* (D) mRNAs using the ^32^P-labelled primers lpp26, ompA-ctr-ccw and rpoD PE1 respectively. Total RNA was prepared from cells of MG1655Δ5 (lacks the toxin antitoxin systems *relBE*, *yefM/yoeB*, *dinJ/yafQ*, *mazEF* and *chpSB*) or MG1655Δ5 carrying on of the plasmids pMG3323 (pBAD::*relE*), pMCD3306 (pBAD::SD::*yafO*), pBAD3310 (pBAD::SD::*higB*) or pMCD3312 (pBAD::SD::*ygiU*) growing exponentially in LB medium at 37°C. At time zero, CML was added to MG1655Δ5 and transcription of the toxin genes from the remaining cells induced with arabinose (0.2%). Cleavage sites mediated by RelE (•), YafO (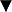), HigB (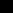) and YgiU (⋄) are marked on the gels and in the relevant RNA sequences below each gel. The numbers above lanes are the time points of cell sampling relative to toxin induction or addition of CML.

**Fig. 4:**
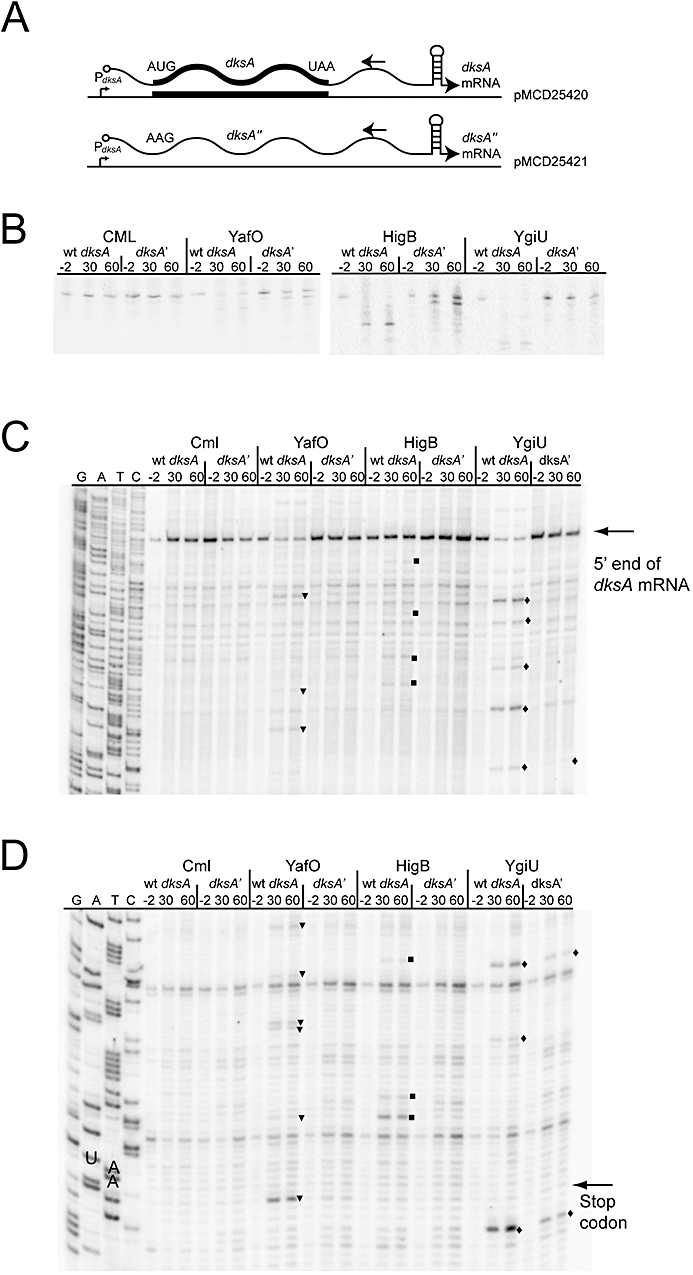
Translation affects target mRNA cleavage differentially. A. Plasmid pMCD25420 produces wild type *dksA* mRNA whereas pMCD25421 produces a *dksA* mRNA with the ATG start codon changed to AAG (*dksA′* mRNA). B–D. The strains MG1655Δ5Δ*dksA*/pMCD25420 or MG1655Δ5Δ*dksA*/pMCD25421 were cotransformed with the plasmids, pMCD3306, pMCD3310 and pMCD3312. Cells were grown exponentially in LB and induced with arabinose (0.2%) at time zero. Total RNA was purified and used for Northern blot analysis using an RNA probe complementary to the *dksA* mRNA (B) and primer extension analysis on *dksA* mRNA using the ^32^P-labelled primer pKW71D-3#PE (C and D), where (C) is a representation of the dksA 5′ end and panel D a representation of the RNA region close to the dksA stop codon. Numbers are time points of cell sampling relative to the addition of arabinose or CML. Significant cleavage sites are indicated with black symbols and important sites in the mRNAs are indicated with arrows to the right.

We measured translation rates before and after induction of the toxin genes. Indeed, ectopic expression of the three toxins led to a rapid reduction of the global rates of translation in all three cases (Fig. 2E). To investigate if this inhibition of translation was caused by mRNA cleavage, model RNAs were monitored using Northern blot and Primer extension analyses. For comparison, RelE of *E. coli* was included in the analysis. Ectopic expression of any of the three toxins induced a rapid degradation of four different mRNAs, *lpp*, *ompA*, *rpoD* (Fig. 3) and *dksA* (Fig. 4). Induction of all three toxins resulted in unique and significant cleavage sites in the model mRNAs. YafO and HigB mediated cleavage patterns clearly resembling that of RelE. Thus, both mRNA interferases mediated cleavages that were confined to the coding regions of the RNAs. Moreover, the most abundant cleavage sites were located between the second and the third bases of the codons, rather than recognizing specific sequences in the RNAs.

The YgiU/MqsR-mediated cleavage sites were scattered over the entire RNAs, although primarily found within coding regions. Cleavage site selection by YgiU/MqsR did not seem to be dependent on the codons but were in all cases observed after C in GC(A/U) sequences. Thus, YgiU/MqsR is most likely a sequence-specific ribonuclease.

### YafO and HigB depend on translation whereas YgiU*/*MqsR does not

To more directly investigate the effect of translation of the model RNAs, we analysed the cleavage patterns of translated and non-translated versions of the *dksA* mRNA after toxin gene induction (the AUG start codon was changed to AAG). These RNAs have previously been used for this type of analysis (Christensen-Dalsgaard and Gerdes, 2008; Winther and Gerdes, 2009). A schematic of the two model mRNAs is presented in Fig. 4A. The level of the wild-type *dksA* mRNA decreased rapidly after toxin gene induction (Fig. 4B). By contrast, the level of the non-translated version of the *dksA* mRNA was not seriously affected by expression of any of the toxins (*dksA′* in Fig. 4B).

Next, we investigated the cleavage patterns of the 5′ and 3′ ends of the two model mRNAs using primer extension analysis (Fig. 4C and D respectively). As seen, induction of *yafO* and *higB* mediated cleavages in the translated version of the *dksA* mRNA whereas no such cleavages were apparent in the non-translated RNA. By contrast, YgiU/MqsR cleaved both versions of the *dksA* mRNA, although the translated RNA appeared to be cleaved more efficiently. Most significantly, *ygiU* induction mediated cleavage in both mRNAs in a GCU sequence located downstream of the *dksA* stop codon, indicating that YgiU/MqsR cleavage occurred independently of translation *in vivo*. In summary, these analyses show that YafO and HigB/YgjN are mRNA interferases that depend on translation whereas YgiU/MqsR is a translation-independent mRNA interferase. Hence, YgiU/MqsR is the first known RelE-like mRNA interferase that cleaves mRNA independently of the ribosome, *in vivo*.

### The three TA loci are transcribed from single promoters that are autoregulated by their cognate antitoxins

The promoters of the three TA loci were mapped using primer extension analysis. RNA was purified from cells exposed to chloramphenicol (CML) that induces strong transcription of previously characterized TA loci (Christensen *et al.*, 2001; Hazan *et al.*, 2004; Christensen-Dalsgaard and Gerdes, 2006). Specific bands consistent with transcriptional start sites were detected upstream of the first gene of all three TA loci (Fig. 5A–C, left). There were no such bands upstream of the second genes, indicating that all three TA loci were transcribed as operons (data not shown). In all three cases, inspection of the DNA sequences upstream of the +1 sites revealed the presence of putative −35 and −10 boxes, reinforcing that the observed bands represented the 5′ ends of the TA-encoding mRNAs (Fig. 1B). The amounts of the mRNA 5′ ends increased dramatically after the addition of CML in all three cases, indicating that the three promoters are regulated by mechanisms similar to that of previously characterized TA operons (see *Discussion*). To test the validity of the proposed promoters, we made operon fusions to *lacZ*. When the fusion plasmids were transformed into TB28Δ*lacIZYA* that encode the cognate antitoxins, transcription remained repressed (data not shown). However, when the fusion plasmids were transformed into strains lacking the cognate TA locus, the promoters were active (Fig. 5A–C, right), showing that all three promoters are negatively autoregulated by the TA operon product(s). As all three antitoxins have DNA binding motifs, we propose that the antitoxins inhibit transcription via binding to their cognate promoter regions. Indeed, ectopic expression of the antitoxins from high-copy plasmids could efficiently inhibit transcription from all three promoters (Fig. 5A–C, right). We conclude that all three TA loci analysed here contain an upstream promoter, which is negatively regulated by the antitoxin.

### Lon protease is required for transcriptional activation of all three TA loci

As Lon has been reported to degrade antitoxins (Van Melderen *et al.*, 1994; Christensen *et al.*, 2001; 2004), we analysed the transcriptional patterns after addition of CML in *lon*, *clp* and *lon clp* strains (Fig. 5). As seen, transcriptional activation was highly reduced in the *lon* strain, whereas deletion of *clp* reduced transcription only slightly. Deletion of both *lon* and *clp* almost completely abolished transcriptional activation of the TA loci. These results show that the protease Lon, and to a lesser extent Clp, is essential for the activation of the three new TA loci.

### Differential induction of the three TA loci by environmental stress

**Fig. 6:**
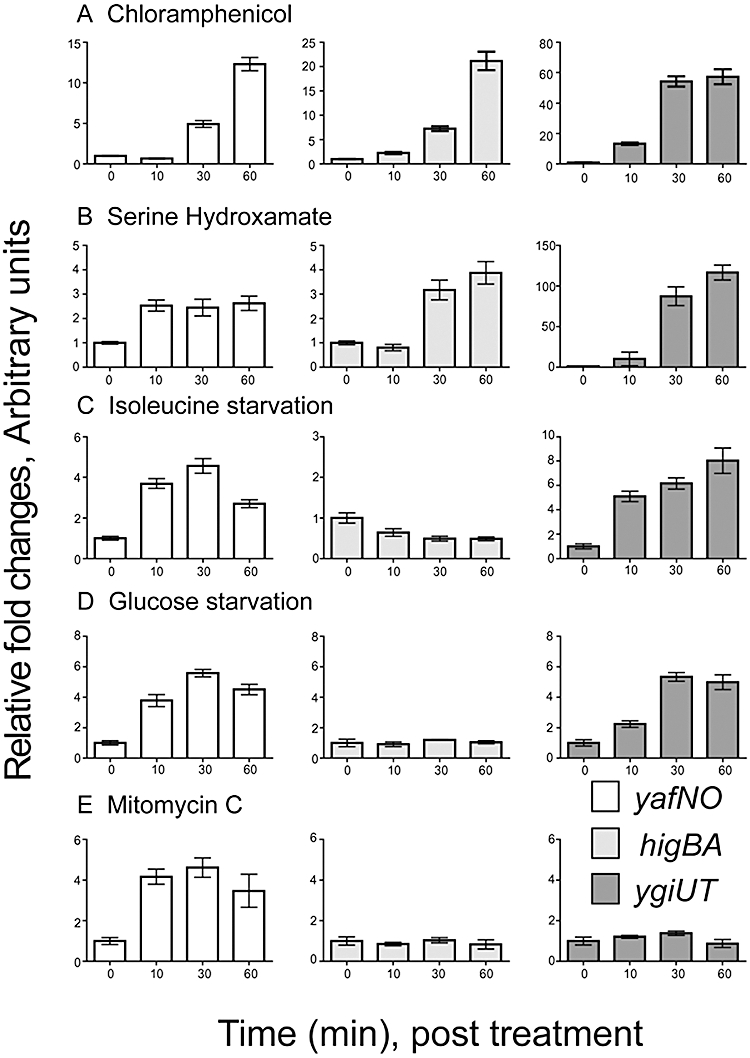
Nutritional stresses differentially induce TA loci transcription. Cells of MG1655 were grown exponentially in M9 minimal medium at 37°C. Samples were taken before and after induction of various stress conditions at the time points indicated. Transcriptional activation of *yafNO, ygiUT and, higBA* (*ygjNM)* were analysed by reverse transcription qPCR and represented by relative fold of changes. (A) 30 μg ml^−1^ CML; (B) 0.5 mg ml^−1^ valine addition; (C) 1% methyl-α- D-glycopyranoside; (D) 0.4 mg ml^−1^ serine hydroxamate; (E) 1 μg ml^−1^ mitomycin C. Note that the different panels have different scales on their Y-axes.

The TA loci have been reported to be activated by several stressful conditions (Christensen *et al.*, 2001; Jørgensen *et al.*, 2009). Here, we used quantitative PCR (qPCR) to measure the relative changes of the levels of the *yafNO*, *higBA* and *ygiUT* mRNAs during conditions of environmental stresses (Fig. 6A–E). In accordance with Fig. 5, CML induced strong transcription of all three gene loci (Fig. 6A). Induction of aa starvation by SHX also induced strong transcription of all three loci (Fig. 6B). Interestingly, however, isoleucince starvation (induced by the addition of valine) induced the *yafNO* and *ygiUT* loci only, whereas the transcription rate of *higBA* actually decreased slightly (Fig. 6C). Glucose starvation activated transcription of *yafNO* and *ygiUT*, whereas *higBA* transcription was not affected in this case (Fig. 6D).

### The *yafNO* locus belongs to the SOS regulon of *E. coli*

By inspection of the *yafNO* promoter sequence, we found that it contains a putative LexA binding site that overlaps with the transcriptional start site and the −10 box (Fig. 1). This is the usual location of functional LexA boxes. Furthermore, the *yafNO* locus has previously been reported to be part of the LexA-regulated *dinB* operon (McKenzie *et al.*, 2003). LexA is responsible for the transcriptional repression of ≈40–60 genes in *E. coli* that are induced in response to DNA damage (the SOS response) (Fernandez De Henestrosa *et al.*, 2000; Wade *et al.*, 2005). We therefore investigated the transcriptional response of the three TA loci after addition of mitomycin C, an inducer of the SOS response. Strikingly, the *yafNO* mRNA level increased in response to mitomycin C, whereas the *higBA* and *ygiUT* mRNAs exhibited no such response (Fig. 6E). However, we speculated if the increased *yafNO* levels could originate from increased activity of the *dinB* promoter located upstream of *yafNO* rather than from the proximal *yafNO* promoter. To test this, we followed the *yafNO* mRNA levels after mitomycin C treatment in a strain in which the *dinB* promoter was deleted. The result clearly showed that the level of *yafNO* mRNA was completely unchanged after mitomycin treatment in the strain lacking the *dinB* promoter (Fig. S2). Thus, the *yafNO* locus is transcribed from two promoters: one SOS-regulated promoter upstream of *dinB* and one immediately upstream of *yafN*, which is autoregulated by the antitoxin YafN.

## Discussion

Here, we characterized three new mRNA interferases of *E. coli* (Figs 2–4). Comparison of their secondary structures with those of other mRNA interferases of *E. coli* clearly indicates that all three mRNA interferases exhibit structural similarity with the RelE family of toxins (Fig. S1). The primary sequences of the RelE family of proteins are highly divergent (Gerdes, 2000; Pandey and Gerdes, 2005). For example, blast searches with *E. coli* RelE as a query does not identify YoeB as a RelE homologue, yet the 3D folds of the two proteins are very similar (Kamada and Hanaoka, 2005; Takagi *et al.*, 2005; Francuski and Saenger, 2009; Li *et al.*, 2009). Combined with the biological data presented here, this similarity strongly indicates that the three new mRNA interferases all belong to the RelE family of mRNA interferases (Fig. S1). During the final stage of this work, *yafNO* and *ygiUT* were confirmed to be TA loci (Yamaguchi *et al.*, 2009; Zhang *et al.*, 2009), thus confirming this conjecture.

In a genomic screen, the *yafNO* locus was previously identified as a putative TA locus, but the cellular target of YafO was not identified (Brown and Shaw, 2003). While this work was ongoing, YafO was described as a ribosome-dependent mRNA interferase (Zhang *et al.*, 2009). This conclusion is in accordance with our observation that YafO activity depends on translation of the target mRNA (Fig. 4).

The *ygiU/mqsR (*motility quorum-sensing regulator) gene has been described as encoding a factor that positively regulates *qseBC* (Gonzalez Barrios *et al.*, 2006), a two-component system that upregulates flagella and motility genes during quorum sensing (Sperandio *et al.*, 2002). The *ygiU/mqsR* mutant strain had a Tn5 element inserted into the *ygiU/mqsR* gene, but it was not established at which level (transcriptional or post-transcriptional) the mutation affected *qseBC* regulation and the fact that YgiU/MqsR is an mRNA interferase thus raises the possibility that the effect was indirect (Gonzalez Barrios *et al.*, 2006). Together with other known TA loci, *ygiU/mqsR* also has been found to be significantly upregulated in persister cells (Shah *et al.*, 2006).

Messenger RNA cleavage by two of the toxins, YafO and HigB/YgjN depended on translation of the target RNAs (Fig. 3). Both toxins cleaved mRNA in a pattern similar to that of RelE (Fig. 4). By contrast, YgiU/MqsR cleaved target RNAs specifically at GC[A/U] sites, independently of translation (Figs 3 and 4), confirming recently published results by Inouye and coworkers (Yamaguchi *et al.*, 2009). In both these properties, YgiU/MqsR resembled MazF and ChpB toxins (Zhang *et al.*, 2003; 2005b).Thus, YgiU/MqsR is, to our knowledge, the first RelE-homologous mRNA interferase that can cleave target RNA independently of translation *in vivo*. However, it should be noted that translation of the target mRNA stimulated its cleavage by YgiU/MqsR, a property that has also be described in the case of MazF (Christensen-Dalsgaard and Gerdes, 2008) (Fig. 4).

All three new TA loci were transcribed from single promoters upstream of the first gene in the operon, all of which were autoregulated by the cognate antitoxins (Figs 1 and 6). Activation of the promoters in all cases depended on Lon protease and to a lesser extent Clp (Fig. 5). These observations are consistent with the finding that the antitoxins YafN, HigA and YgiT autoregulate transcription of their cognate TA loci (via binding to the promoter regions) and are degraded by Lon. Thus, transcriptional activation of the three operons by CML and SHX can be explained by drug-induced decay of the antitoxins followed by derepression of the TA promoters.

All three TA promoters were activated by nutritional stresses but, interestingly, they responded differently to different stresses (Fig. 6). All three loci were induced by ‘strong’ aa starvation (addition of SHX induces ‘strong’ aa starvation). The *yafNO* and *ygiUT* loci were also activated by mild aa starvation (induced by the addition of valine), and these two loci were also induced by glucose starvation. *E. coli* encodes at least 10 TA loci (Fig. 1A), all of which are induced by aa starvation (Christensen *et al.*, 2001; 2003; Jørgensen *et al.*, 2009; this work). One working hypothesis is that TA loci are stress response elements that are activated during unfavourable growth conditions to reduce translation and thereby somehow mitigate the detrimental effects conferred by the stress. It is also possible, and not mutually exclusive with the stress hypothesis, that mRNA interferases function to rapidly reprogram the ribosomes with newly synthesized mRNA after sudden changes in growth conditions – in other words, mRNA interferases may function ‘to wipe the board clean and start all over’. We are now testing these two hypotheses.

Interestingly, mitomycin C induced *yafNO* but not the two other TA loci (Fig. 6). The *yafNO* locus is located just downstream of *dinB*, another SOS-induced gene important for error-prone DNA repair by transletion synthesis of DNA (McKenzie *et al.*, 2001) and is probably cotranscribed with *dinB* by an SOS-induced promoter (McKenzie *et al.*, 2003). We identified a putative SOS box in the *yafNO* promoter (Fig. 1B), but our results clearly showed that the transcriptional induction of *yafNO* in response to mitomycin C originated from the upstream *dinB* promoter rather than the proximal *yafNO* promoter (Fig. S2). The observation that *dinB-yafNO-dinP* is induced by DNA damage raises the possibility that mRNA cleavage might be advantageous during conditions of DNA damage stress. This proposal is supported by the fact that *E. coli* contains at least two additional SOS-induced mRNA interferases, *symE* and *dinJ yafQ* (Kawano *et al.*, 2007; Prysak *et al.*, 2009). Interestingly, *E. coli* codes for additional TA-encoded toxins belonging to the SOS regulon, TisB and HokE (Fernandez De Henestrosa *et al.*, 2000; Vogel *et al.*, 2004). Expression of these latter two genes is regulated by small antisense RNAs. The *yafNO* locus is also autoregulated by the TA complex as CML and SHX induced strong transcription of the locus (Figs 5 and 6). Thus, the coupling to the SOS response adds to the complexity of the regulation of this TA operon.

Here we have identified three novel TA loci of *E. coli* K-12 and shown that they encode mRNA interferases related to RelE. The TA genes were all induced by strong aa starvation but responded differentially to other types of metabolic and environmental stress. Given these differences and similarities with the other mRNA interferase-encoding TA loci of *E. coli*, we believe that it will be essential to include these three new TA loci in a meaningful analysis of the physiological effects of TA loci.

## Experimental procedures

### Bacterial strains and plasmids

All bacterial strains and plasmids used in this work are listed in Table 1. Strains and plasmids that were constructed specifically for this work are described below. Oligonucleotides are listed in Table S1.

**Table 1:** Strains and plasmids.

Strains/plasmids	Genotype/plasmid properties	References
Strains		
MG1655	Wild-type *E. coli* K12	
TB28	MG1655Δ*lacIZYA*	Bernhardt and de Boer (2003)
BW25113	lacI^q^*rrnBT*14 ΔlacZ_WJ16_*hsdR*514 Δ*araBAD*_AH33_Δ*rhaBAD*_LD78_	Datsenko and Wanner (2000)
AG1	*endA*1 *recA*1 *gyr*A96 *thi*-1 *relA*1 *gln*V44 *hsdR*17(r_K_^-^ m_K_^+^)	Kitagawa *et al.* (2005)
MG1655Δ*dksA*::*tet*	MG1655Δ*dksA*::*tet*	Christensen-Dalsgaard and Gerdes (2008)
SC301467	MG1655 ΔmazF ΔchpSB Δ*relBE*Δ (dinJ-yafQ) Δ (yefM-yoeB)	Christensen *et al.* (2004)
JW0223	BW25113Δ*yafN*::*kan*	Baba *et al.* (2006)
JW3054	BW25113Δ*ygjN*::*kan*	Baba *et al.* (2006)
JW2990	BW25113Δ*ygiU*::*kan*	Baba *et al.* (2006)
BW25113ΔP_*dinB*_::cat	BW25113ΔP_*dinB*_::cat	This work
SC301467Δ*dksA*::*tet*	SC301467Δ*dksA*::*tet*	This work
MG1655Δ*ygjN*::*kan*	MG1655Δ*ygjN*::*kan*	This work
MG1655Δ*ygiU*::*kan*	MG1655Δ*ygiU*::*kan*	This work
MCD2801	TB28Δ*yafNO*::*kan*	This work
MCD2802	TB28Δ*ygjNM*::*kan*	This work
MCD2803	TB28Δ*ygiUT*::*kan*	This work
MCD03	MG1655 ΔP_*dinB*_::*cat*	This work
Plasmids		
pBAD33	p15; *cat araC* pBAD	Guzman *et al.* (1995)
pMG3323	pBAD33; pBAD::*relE*	Christensen and Gerdes (2003)
pMCD3306	pBAD33; pBAD::SD::*yafO*	This work
pMCD3310	pBAD33; pBAD::SD::*higB* (*ygjN*)	This work
pMCD3312	pBAD33; pBAD::SD::*ygiU*	This work
pNDM220	R1; *bla lacI*^q^ pA1/O4/O3	Gotfredsen and Gerdes (1998)
pMCD2202	pNDM220; pA1/O4/O3::SD::*yafN*	This work
pMCD2205	pNDM220; pA1/O4/O3::SD::*ygjM*	This work
pMCD2207	pNDM220; pA1/O4/O3::SD::*ygiT*	This work
pKW254T	R1; terminator from pMG25	Winther and Gerdes (2009)
pMCD25420	R1; pKW254T *dksA*	Christensen-Dalsgaard and Gerdes (2008)
pMCD25421	R1; pKW254T *dksA*′	Christensen-Dalsgaard and Gerdes (2008)
pOU254	R1; *bla par mcs-lacZYA*	R.B. Jensen, unpubl. data
pMCD25433	R1; pOU254::P_*yafNO*_	This work
pMCD25434	R1; pOU254::P_higBA_	This work
pMCD25435	R1; pOU254::P_*ygiUT*_	This work
pCA24N	Cm^R^; *lacI*^q^, pCA24N	Kitagawa *et al.* (2005)
pCA24N::*yafN*	pCA24N P_T5-lac_::*yafN*	Kitagawa *et al.* (2005)
pCA24N::*ygjM*	pCA24N P_T5-lac_::*ygjM*	Kitagawa *et al.* (2005)
pCA24N::*ygiT*	pCA24N P_T5-lac_::*ygiT*	Kitagawa *et al.* (2005)
pSC333	pGEM3, *bla*, T_7_::*lpp*	Christensen and Gerdes (2003)

### Strains and plasmids constructed

#### 

##### 

###### MCD03

The strain (MG1655 ΔP_*dinB*_::*cat*) was constructed as described by Datsenko and Wanner (2000). A PCR product was made with pKD3 template and the following primers delta dinB-cw and delta dinB-ccw1. The PCR product was electroporated into BW25113/pKD46 and the cells were spread on LB plates containing 25 μg ml^−1^ of CM and incubated ON at 37°C. The deletion of dinB promoter was verified by PCR. A P1 lysate was made from BW25113 ΔP_*dinB*_::*cat* and the *cat* allele was transduced into MG1655.

###### MCD2801

A P1 lysate was made from MG1655Δ*yafNO*::*kan* (M.G. Jørgensen, unpublished) and the *kan* allele was transduced into TB28.

###### MCD2802

A P1 lysate was made from MG1655Δ*higBA*::*kan* (M.G. Jørgensen, unpublished) and the *kan* allele was transduced into TB28.

###### MCD2803

A P1 lysate was made from MG1655Δ*ygiUT*::*kan* (M.G. Jørgensen, unpublished) and the *kan* allele was transduced into TB28.

###### pMCD3306

The *yafO* gene was amplified from chromosomal DNA of MG1655 with primers yafO-XbaI-SD-up and yafO-HindII-down. The PCR product was digested with XbaI and HindIII and inserted into pBAD33. The resulting plasmid contains the *yafO* gene with an efficient SD sequence downstream of the P_BAD_ promoter.

###### pMCD3310

The *higB (ygjN*) gene was amplified from chromosomal DNA of MG1655 with primers ygjN-XbaI-SD-cw and ygjN-HindII-ccw. The PCR product was digested with XbaI and HindIII and inserted into pBAD33. The resulting plasmid contains the *higB (ygjN*) gene with an efficient SD sequence downstream of the P_BAD_ promoter.

###### pMCD3312

The *ygiU* gene was amplified from chromosomal DNA of MG1655 with primers msqR-cw and msqR-ccw. The PCR product was digested with XbaI and HindIII and inserted into pBAD33. The resulting plasmid contains the *ygiU* gene with an efficient SD sequence downstream of the P_BAD_ promoter.

###### pMCD2202

The *yafN* gene was amplified from chromosomal DNA of MG1655 with primers yafN-KpnI-SD-up and yafN-SalI-down. The PCR product was digested with BamHI and XhoI and inserted into pNDM220. The resulting plasmid contains the *yafN* gene with an efficient SD sequence downstream of the pA1/O4/O3 promoter.

###### pMCD2205

The *ygjM* gene was amplified from chromosomal DNA of MG1655 with primers ygjM-KpnI-SD-cw and ygjM-XhoI-ccw. The PCR product was digested with KpnI and XhoI and inserted into pNDM220. The resulting plasmid contains the *ygjM* gene with an efficient SD sequence downstream of the pA1/O4/O3 promoter.

###### pMCD2207

The *ygiT* gene was amplified from chromosomal DNA of MG1655 with primers ygiT-cw and ygiT-ccw. The PCR product was digested with BamHI and XhoI and inserted into pNDM220. The resulting plasmid contains the *ygiT* gene with an efficient SD sequence downstream of the pA1/O4/O3 promoter.

###### pMCD25433

The region upstream of the *yafNO* locus was amplified with primers yafN-105-cw and yafN-ccw. The PCR product was digested with EcoRI and BamHI and inserted into pOU254. The resulting plasmid contains a transcriptional fusion of the *yafNO* promoter with *lacZ*.

###### pMCD25434

The region upstream of the *higBA (ygjNM*) locus was amplified with primers ygjN-124-cw and ygjN-ccw2. The PCR product was digested with EcoRI and BamHI and inserted into pOU254. The resulting plasmid contains a transcriptional fusion of the *higBA* promoter with *lacZ*.

###### pMCD25435

The region upstream of the *ygiUT* locus was amplified with primers mqsR-109-cw and mqsR-ccw2. The PCR product was digested with EcoRI and BamHI and inserted into pOU254. The resulting plasmid contains a transcriptional fusion of the *ygiUT* promoter with *lacZ*.

### Growth conditions and media

Cells were grown in either Luria–Bertani broth or M9 minimal medium supplemented with aa in defined concentrations and 0.2% glucose or 0.5% glycerol at 37°C. When appropriate, the medium was supplemented with ampicillin (30 μg ml^−1^), CML (50 μg ml^−1^), kanamycin (25 μg ml^−1^) or tetracycline (10 μg ml^−1^). When aa starvation was induced in M9 minimal medium by the addition of 0.4 mg ml^−1^ SHX (Sigma-Aldrich), serine was excluded from the medium. Glucose starvation was induced in M9 minimal medium containing 0.05% glucose by the addition of 1% methyl-α-D-glucopyranoside (Sigma-Aldrich). Isoleucine starvation was induced in M9 minimal medium by addition of 0.5 mg ml^−1^ valine. The SOS response was induced in M9 minimal medium by addition of 1 μg ml^−1^ mitomycin C. Expression of the P_A1/O4/O3_ promoter was induced by the addition of 2 mM IPTG and expression of the P_BAD_ promoter was induced by the addition of 0.2% arabinose.

### Rates of protein synthesis

Cells were grown at 37°C in M9 minimal medium + 0.5% glycerol and amino acids in defined concentrations to an optical density (OD_450_) of 0.5. The cultures were diluted 10 times and arabinose was added at an OD of 0.3. Samples of 0.5 ml were added to 5 μCi of [^35^S]methionine and after 1 min of incorporation, samples were chased for 10 min with 0.5 mg of cold methionine. The samples were harvested and resuspended in 200 μl cold 20% trichloracetic acid and were centrifuged at 20 000 *g* for 30 min at 4°C. The samples were washed twice with 200 μl cold 96% ethanol. Precipitates were transferred to vials and the amount of incorporated radioactivity was counted in a liquid scintillation counter.

### Northern blot and primer extension analysis

Cells were grown in Luria–Bertani medium at 37°C. At an OD_450_ of 0.5, the cultures were diluted 10 times and grown to an OD of 0.5 and transcription of the toxin genes was induced by the addition of 0.2% arabinose. To inhibit translation, CML (50 μg ml^−1^) was added. For Northern analysis, total RNA was fractionated by PAGE (6% low-bis acrylamide), blotted to a Zeta-probe nylon membrane and hybridized with a single-stranded ^32^P-labelled riboprobe complementary to the RNA of interest. For *lpp* mRNA hybridization, the radioactive probe was generated by T7 RNA polymerase using linearized plasmid DNA of pSC333 as the template. The ribopropes used to detect *dksA* and *ompA* mRNA were transcribed from a PCR fragments containing a complementary region of the *dksA* (constructed using the primers *dksA* probe-f and *dksA* T7 probe-r) and ompA (constructed using the primers T7/pmoA and pmoA-forw) genes. Primer extension analysis was used to map promoters and the cleavage patterns of the *lpp*, *ompA*, *rpoD* and *dksA* mRNAs, using ^32^P-labelled primers, following extension by reverse transcriptase. The primer (3 pmol) was labelled with 2 μl of [γ-^32^P]-ATP at a concentration of 6000 Ci mmol^−1^ by addition of 0.4 μl polynucleotide kinase (New England Biolabs) in polynucleotide kinase buffer and incubated for 1 h at 37°C. Labelled primer was hybridized to 10–20 μg total RNA and extended with reverse transcriptase (SuperScript II; Invitrogen). The labelled cDNA was fractionated by using a 6% polyacrylamide gel electrophoresis, which was dried and placed on a PhosphorImager screen.

### Reverse transcription qPCR

RNA was extracted from all cell samples using RNeasy mini-kit (Qiagen) according to the manufacturer's instructions. cDNA synthesis was performed using a high-capacity cDNA reverse transcription kit (Applied Biosystems). qPCR reactions were run in triplicates simultaneously. Briefly, 10 ng cDNA was mixed with 0.3 μM primers and 10 μl of 2× PCR Master Mix for Sybr green kit from Eurogentec (Seraing, Belgium), and the qPCR was run on a LightCycler 480 real-time PCR system (Roche). Relative folds of expression were calculated using the qBase software using *tatA* and *eutA* as reference genes (Hellemans *et al.*, 2007).
