# Supplementary material for: Three new RelE-homologous mRNA interferases of Escherichia coli differentially induced by environmental stresses
Source: Mol Microbiol. 2009 Dec 3;75(2):333–48. doi: 10.1111/j.1365-2958.2009.06969.x (PMC2814082; doi:10.1111/j.1365-2958.2009.06969.x)
Supplement: Supplementary file 1 [file mmi0075-0333-SD1.pdf]

Supplementary Information **to Christensen-Dalsgaard et al.. 2010.**

Legends to supplementary Figures

Figure S1. Secondary structure comparison of the seven known and three new mRNA interferases of E. coli K-12. The secondary structures were predicted from amino acid sequences using the online tool PSIPRED (McGuffin *et al.*, 2000). The comparison indicate that the 10 mRNA interferases belong to three different gene families. Together with the biological data, the secondary structures show that the three new toxins, YafO, HigB/YgjN and YgiU/MqsR all belong to the RelE super-family.

Figure S2. Transcriptional activation of *yafNO* during mitomycin C treatment is dependent on the *dinB* promoter.
Cells of MG1655 and MCD03 (MG1655ΔP_{dinB}::cat) were grown exponentially in M9 minimal medium at 37°C. Samples were taken before and after Mitomycin C treatment (1 ug/ml) at the time points indicated. Transcriptional activation of *yafNO* was analysed by reverse-transcription quantitative PCR and represented by relative fold-of-changes. Note that the different panels have different scales on their Y-axes.

Table S1. DNA oligonucleotides used (shown 5' to 3')

Oligonucleotide Name	Sequence
yafO-XbaI-SD-up	CCCCCTCTAGAGTCGACTCAAGGAGTTTTATAAATGCGGGTATTCAAAACAAAAC
yafO-HindII-down	CCCCCAAGCTTCATAATATAAATTCAAAAACGCATG
ygjN-XbaI-SD-cw	CCCCCTCTAGAGTCGACTCAAGGAGTTTTATAAATGCACCTGATAACTCAAAAAG
ygjN-HindII-ccw	CCCCCAAGCTTGCAATCATTTTTTCCCCTTAG
msqR-cw	CCCCCTCTAGAGGATCCTCAAGGAGTTTTATAAATGGAAAAACGCACACCACAT
msqR-ccw	CCCCCAAGCTTCTCGAGCATATTACTTCTCCTTAAACGAG
yafN-KpnI-SD-up	CCCCCGGTACCGGATCCAAAAGGAGGAAAAAACCATGCATCGAATTCTCGCTGAA A
yafN-SalI-down	CCCCCCTCGAGGTCGACCCGCATCCTTATTCCTTAAAG
ygjM-KpnI-SD-cw	CCCCCGGTACCGAATTCAAAAGGAGGAAAAAACCATGATTGCGATTGCCGACATC
ygjM-XhoI-ccw	CCCCCCTCGAGGGATCCGTTAATCAATAAACAAGGCGGG
ygiT-cw	CCCCCGGATCCTCTAGATCAAGGAGTTTTATAAATGAAATGTCCGGTTTGCCAC
ygiT-ccw	CCCCCCTCGAGAAGCTTCCATTAATTAACGGATTTCATTC
yafN-105-cw	CCCCGAATTCCATGTGACGTTGCTTGACCC
yafN-ccw	CCCCGGATCCGATGCATACAGTGATACCCTC
ygjN-124-cw	CCCCGAATTCGTCTACATTCTCTTGTTTAGCG
ygjN-ccw2	CCCCGGATCCGTGCATTCTTCATCACGTCC
mqsR-109-cw	CCCCGAATTCCGTTTTGTGTGGTCACTATCTC
mqsR-ccw2	CCCCGGATCCTGCGTTTTTCCATAACCCCC
yafN PE1	CGTAACTCAGTGATATTGACC
ygjN PE1	CCGCAGCATCTTTCAATGC
ygiU/mqsR PE1	CTTGCCCGGCATTGACAAG
lpp 26	CAGCTGGTCAACTTTAGCGTTCAGAG
ompA-ctr-ccw	GGTATTCCAGACGGGTAGCG
rpoD PE1	GATTCCACGCTGGAAAGCAC
dksA PE1	GATATGGTTCCACCCCAGCG
pKW71D-3#PE	GAGGTCATTACTGGATCTATCAAC
dksA probe– f	CAACTTCCCGGACCCGGTAG
dksA T7 probe- r	GGGCCTAATACGACTCACTATAGGGGATGCACAGATC GGCTGTCG
T7/pmoA	GGGCCTAATACGACTCACTATAGGG ACCAGGTGTTATCTTTCGGAGCGGCCTGCGC
pmoA-forw	CGGCATAAGCCGAAGATATCGG
delta dinB-cw	GCAAAAGCTGGATAAGCAGCAGGTGCTTTCGCAGCGAACGCGTTAAATGCGTGT AGGCTGGAGCTGCTTC
delta dinB-ccw1	CACTGCGGCGAAAAAGCAGTCCATATCCACATGAATGATTTTACGCATTGCCATAT GAATATCCTCCTTAG
qPCR-*yafNO*-f	AATAATCCATTTCCGCCACA
qPCR-*yafNO*-r	TCAGAATGGCAATGAGCAAC
qPCR-*ygjNM*-f	TGGGGAACACGATTGCTAA
qPCR-*ygjNM*-r	TAATTCATTGCCCCCAACAT
qPCR-*ygiUT*-f	TGCCCTGTTAAATGCAGATG
qPCR-*ygiUT*-r	GCCTGGGTCTGTAAACATCCT
qPCR-tatA-f	TGAGCGATGATGAACCAAAG
qPCR-tatA-r	TCGCGTCTTCTGTTTTAGCC
pPCR-eutA-f	CTCTCTGGCAGCACAATCTG
qPCR-eutA-r	GCGTCAGTTTTGGGATCAAG

The 10 TA-encoded mRNA interferases of E. coli belong to three different families:

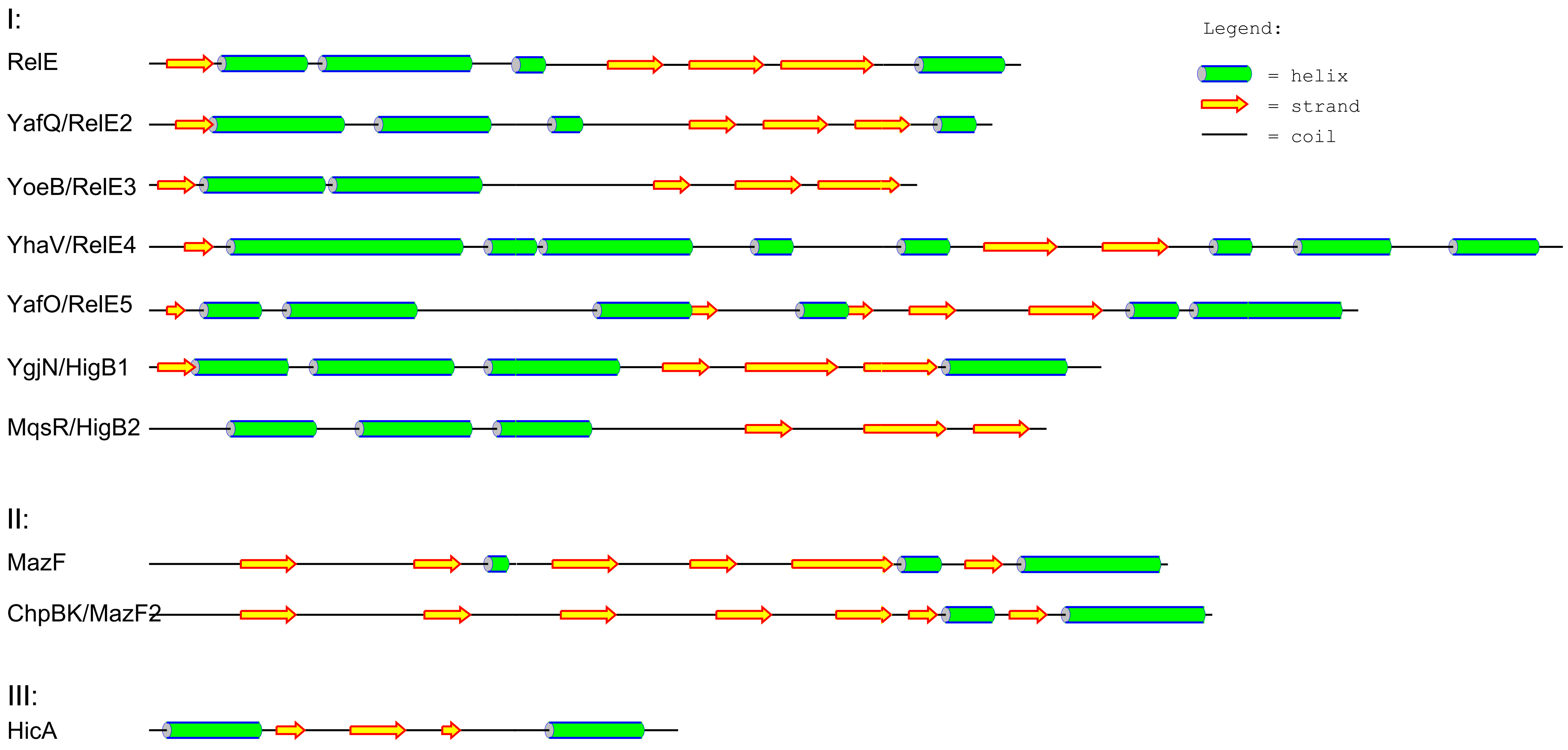

Fig. S1

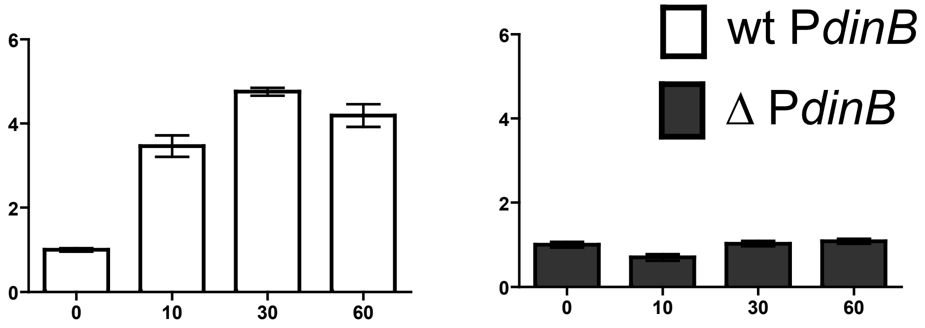

Figure S2
